# Caffeine promotes wakefulness via dopamine signaling in *Drosophila*

**DOI:** 10.1038/srep20938

**Published:** 2016-02-12

**Authors:** Aleksandra H. Nall, Iryna Shakhmantsir, Karol Cichewicz, Serge Birman, Jay Hirsh, Amita Sehgal

**Affiliations:** 1 Cell and Molecular Biology Program, Perelman School of Medicine, University of Pennsylvania, Philadelphia, PA 19104, USA; 2 Genes Circuits Rhythms and Neuropathologies, Brain Plasticity Unit, CNRS, PSL Research University, ESPCI ParisTech, 10 rue Vauquelin, 75005 Paris, France; 3 Department of Biology, University of Virginia, Charlottesville, VA 22904, USA; 4 Howard Hughes Medical Institute, Perelman School of Medicine, University of Pennsylvania, Philadelphia, PA 19104, USA

## Abstract

Caffeine is the most widely-consumed psychoactive drug in the world, but our understanding of how caffeine affects our brains is relatively incomplete. Most studies focus on effects of caffeine on adenosine receptors, but there is evidence for other, more complex mechanisms. In the fruit fly *Drosophila melanogaster*, which shows a robust diurnal pattern of sleep/wake activity, caffeine reduces nighttime sleep behavior independently of the one known adenosine receptor. Here, we show that dopamine is required for the wake-promoting effect of caffeine in the fly, and that caffeine likely acts presynaptically to increase dopamine signaling. We identify a cluster of neurons, the paired anterior medial (PAM) cluster of dopaminergic neurons, as the ones relevant for the caffeine response. PAM neurons show increased activity following caffeine administration, and promote wake when activated. Also, inhibition of these neurons abrogates sleep suppression by caffeine. While previous studies have focused on adenosine-receptor mediated mechanisms for caffeine action, we have identified a role for dopaminergic neurons in the arousal-promoting effect of caffeine.

Caffeine is the most widely consumed psychoactive drug in the world^1^. Its popularity is likely due to its ability to fight drowsiness and promote arousal. In addition, caffeine can reverse the effects of sleep deprivation on alertness and cognition, as shown in both rats and humans^1^,^2^. Despite the ubiquity of caffeine in our food and drinks, our understanding of how caffeine affects our brains and bodies is relatively incomplete.

The most extensively-studied behavioral effect of caffeine is acute locomotor stimulation, which has been attributed to antagonism of adenosine receptors. There are four subtypes of adenosine receptor, and caffeine antagonizes both A2A and A1 receptors *in vivo*^3^. Which of these two receptor subtypes is responsible for the motor-stimulating effect, however, is a point of contention^4^,^5^. The effect of caffeine on sleep has been relatively less well-studied. A2A receptors have been implicated in the acute wake-promoting effect of caffeine^6^,^7^, but adenosine receptors do not have an essential role in driving baseline sleep behavior. A1 and A2A mutant mice, which should mimic receptor antagonism, have no baseline sleep defects^6^,^8^. A brain-specific deletion of the A1 receptor causes reduced slow wave brain activity following sleep deprivation, but these mice do not exhibit a change in the time spent in sleep or wake states^9^. In addition to adenosine receptors, caffeine has many other biological targets, including GABA_A_ receptors, ryanodine receptors, glycine receptors, and phosphodiesterases^10^, some of which could be relevant for its effects on sleep.

We use a potent genetic model, the fruit fly *Drosophila melanogaster*, to understand how caffeine promotes wakefulness. Flies have one known adenosine receptor, *dAdoR*, which only shows 30% sequence similarity to the human adenosine receptors at the amino terminal, but has conserved ligand-binding residues^11^. Surprisingly, the *dAdoR* null mutant responds to caffeine identically to wild type flies, suggesting that caffeine promotes wake in *Drosophila* via adenosine receptor-independent mechanisms^12^. Dopamine receptors have been implicated in the effects of caffeine in *Drosophila*, and may also influence caffeine action in mammals through interactions with adenosine receptors^13^,^14^, but a direct involvement of dopamine has not been addressed. Because the behavioral effects of caffeine are similar between flies and humans, understanding the mode of action in the fly may elucidate novel actions of caffeine in mammals as well.

Here, we show that the wake-promoting effect of caffeine in *Drosophila* requires the synthesis of dopamine, a potent wake-promoting neurotransmitter. The modulation of dopaminergic signaling by caffeine likely occurs presynaptically. In addition, we identify a cluster of dopaminergic neurons, which are essential for the caffeine response. We hypothesize that caffeine promotes wake by increasing activity of these neurons.

## Materials and Methods

### Fly Lines

All flies were raised in vials containing fly food (194g cornmeal, 81.3 g dry yeast, 184.8 g molasses, 24 g agar, 30.5 ml of 20% Tegosept and 7 ml of propionic acid per 3 liters of food). Wild type iso31 flies^15^ were used as a positive control for the effects of caffeine, as they consistently display reduced nighttime sleep after consuming drug-containing food. *DTH BAC* and *DTH*^*FS*±^ *BAC* flies are analogous to the *DTH* whole animal (*DTHg*) and hypoderm selective (*DTHg*^*FS*±^) rescue lines as constructed in Riemensperger *et al.*^16^, but constructed via recombineering and genomic integration utilizing endogenous DTH regulatory elements in place of GAL4/UAS binary expression tools. Details of these strains will be published elsewhere. VMAT^p1^ mutants were a kind gift of the Krantz lab (UCLA, Los Angeles, CA). TH-Gal4 (BSC8848) flies were ordered from the Bloomington Stock Center (Bloomington, IL). The restricted dopaminergic drivers TH-C1-Gal4, TH-D1-Gal4, TH-D4-Gal4, TH-F1-Gal4, TH-F2-Gal4, TH-G1-Gal4 were all generously shared with us by the Wu lab (Johns Hopkins University, Baltimore, MD), and the other dopaminergic drivers InSite0104-Gal4 and InSite0273-Gal4 were shared by the Clandinin lab (Stanford University, Stanford, CA). UAS-Shibire^ts^ flies were a gift of the David Anderson lab (CalTech, Pasadena, CA). UAS-CaLexA flies were a gift of Dr. Jing Wang’s lab (UCSD, La Jolla, CA). UAS-dTrpA1 was a gift from L. Griffith (Brandeis University, Waltham, MA).

### Behavioral Assays

To assay sleep behavior, we used the *Drosophila* Activity Monitoring System (DAMS 3.8, TriKinetics). 5- to 10-day-old flies were individually placed in 5 mm glass tubes containing food composed of 5% sucrose and 2% agar (sucrose/agar food). Following a 1.5-day period of acclimation in incubators kept on a 12:12 light/dark schedule at 25 °C, activity was monitored for five consecutive days. Sleep behavior was analyzed using PySolo software, and sleep bouts were defined at 5 or more minutes of inactivity^17^. For all experiments, 10–16 flies were used per treatment group, sex, and genotype. Sleep graphs depict an average of all 5 days of monitoring across all 10–16 flies. While most experiments were conducted using both male and female flies, PySolo sleep graphs for female flies are shown, except where otherwise specified.

For circadian experiments, flies were entrained in DAMS monitors for two days in 12 hour light-dark cycles and then moved to constant darkness for five days. Circadian rhythms of activity were determined using ClockLab software^18^.

For Shibire^ts^ temperature shift experiments, fly crosses were set and raised at 18 °C to avoid prematurely silencing the neurons. Flies were then loaded into DAMS monitors in incubators set to 12:12 light-dark cycles at a temperature of 21 °C, which is permissive for the Shibire^ts^ mutation. The next day, the temperature increased to 30 °C, the restrictive temperature, at lights-on. Sleep was assayed during three days at 30 °C and averaged across all days.

For TrpA1 temperature shift experiments, fly crosses were set and raised at 18 °C to avoid prematurely activating the neurons. Flies were then loaded into DAMS monitors in incubators set to 12:12 light-dark cycles at a temperature of 21 °C, which does not activate the TrpA1 channel. After 3 days of recording, the temperature was increased to 28 °C, which opens the TrpA1 channel. Sleep was assayed during three days at 28 °C and averaged across all days.

### Drug Feeding

Caffeine (Sigma-Aldrich) was mixed into melted sucrose/agar food at a concentration of 0.5 mg/ml for all experiments except for the dose-response experiment where 0.2, 0.5, and 1 mg/ml were used. L-DOPA (Tocris) was mixed into melted sucrose/agar food at a concentration of 3 mg/ml.

### Confocal Microscopy

The CaLexA tool was used to measure the activity of specific populations of neurons^19^. CaLexA flies express a chimeric transcription factor containing a calcium-sensitive regulatory domain of the transcription factor NFAT and the LexA DNA binding and activation domains. Increases in intracellular calcium cause this transcription factor to enter the nucleus, where it binds to and activates a GFP transgene. 5- to 10-day-old CaLexA flies were moved from vials containing fly food to vials containing either sucrose/agar food or sucrose/agar food with 0.5 mg/ml caffeine. After 24 hours, flies were anesthetized on ice, and brains were dissected in 1× phosphate-buffered saline (PBS) containing 0.1% Triton X-100 (PBS-T). Ten brains were dissected per genotype, and all brains were fixed for 1 hour in 4% paraformaldehyde (PFA; Electron Microscopy Sciences). Brains were washed in PBS-T and blocked for one hour in PBS-T containing 5% normal donkey serum (NDS; Jackson ImmunoRes). Brains were incubated at 4 °C overnight in primary antibody in PBS-T with 5% NDS. CaLexA signal was labeled with 1:1000 dilution of rabbit α-GFP (Life Technologies) and neuropil was stained with a 1:1000 dilution of mouse α-nc82 (Developmental Studies Hybridoma Bank). Brains were washed three times with PBS-T and stained for 2 hours with secondary antibodies in PBS-T with 5% NDS. 1:1000 dilutions were used for α-rabbit AlexaFluor 488 (Invitrogen) and α-mouse AlexaFluor 633 (Invitrogen). Brains were washed three times in PBS-T and mounted on slides using VectaShield (Vector Laboratories, Inc.). Slides were imaged on a Leica SP5 confocal microscope with a 20× objective and 0.5 μm step size. GFP intensities were quantified post-hoc on a cell-by-cell basis from individual Z-planes using ImageJ software^20^ and normalized to background GFP intensity.

## Results

### The behavioral response to caffeine requires dopamine synthesis in *Drosophila*

**Figure 1:**
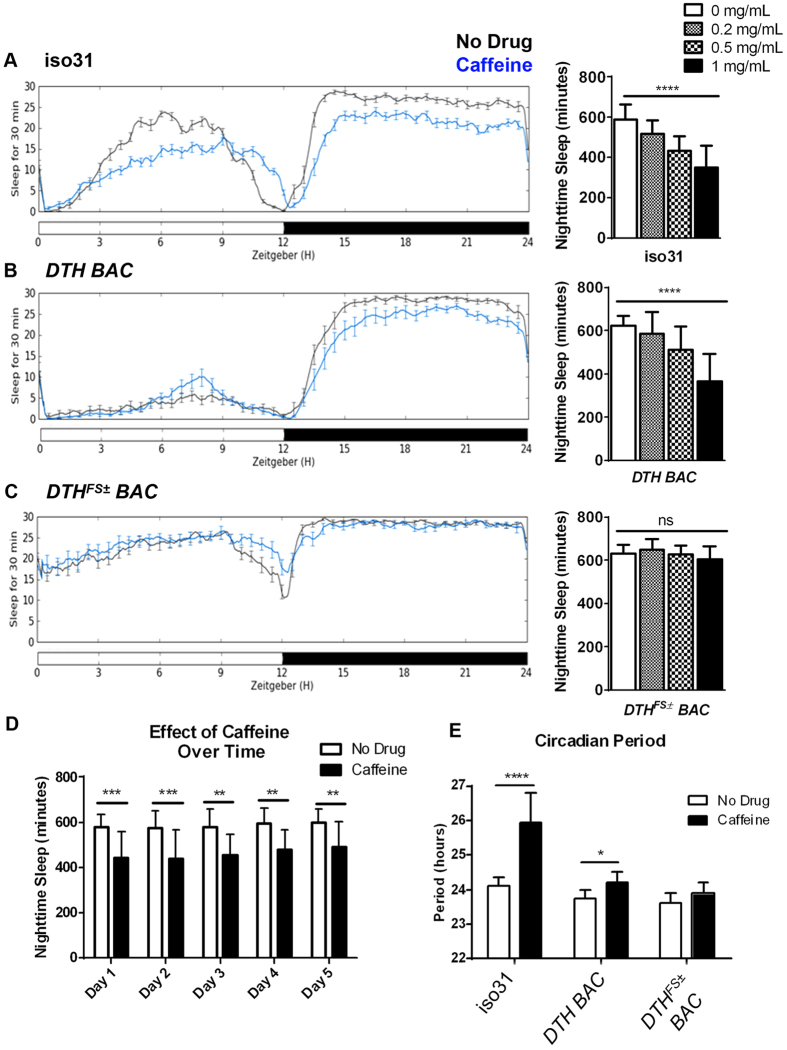
The response to caffeine in *Drosophila* requires dopamine synthesis. Sleep profiles and quantified nighttime sleep for female (**A**) iso31, (**B**) *DTH BAC*, and (**C**) *DTH*^*FS*±^ *BAC* flies. Actograms depict minutes of sleep per 30 minute sliding window across a 24-hour period composed of 12 hours of light (white bar) and 12 hours of dark (black bar). Sleep is shown for flies on drug-free food (black line) or food containing 0.5 mg/ml caffeine (blue line). Bar graphs quantify average number of minutes of sleep per night for flies of each genotype fed either drug-free food or food containing 0.2, 0.5, or 1 mg/ml caffeine. (**D**) The average minutes of nighttime sleep is shown for female iso31 flies fed either drug-free food (white bars) or food containing 0.5 mg/ml caffeine (black bars) during each of the 5 nights of activity monitoring. (**E**) Circadian period of free-running rest-activity rhythms is plotted for the same three genotypes fed drug-free food or food containing 0.5 mg/ml caffeine. Error bars show standard deviation. See text for details of the statistical analysis. Statistical significance thresholds are as follows: * for p < .05, ** for p < .01, *** for p < .001, **** for p < .0001.

Similarly to mammals, *Drosophila* experience reduced sleep following caffeine feeding. This reduction of sleep can be seen in wild type (iso31^15^) flies across a 24-hour light/dark cycle, but the effect is most robust and reproducible in the dark (nighttime) phase (Fig. 1A). Increasing concentrations of caffeine cause further decreases in nighttime sleep, indicating that the effect is dose-dependent (p < 0.0001 for the effect of drug concentration by 2-way ANOVA with Bonferroni multiple comparisons; Fig. 1A). The 0.5 mg/mL caffeine concentration produced the largest, most reproducible loss of sleep with no toxic effects, so this concentration was used for the rest of the experiments reported here. Chronic caffeine feeding caused sustained decreases in nighttime sleep across multiple 24-hour periods (Fig. 1D). During 5 days of activity monitoring, the effect of drug on nighttime sleep was statistically significant (p < 0.0001), while the effect of time was not (p = 0.0724) by two-way ANOVA. Using Bonferroni multiple comparisons, the caffeine-fed flies slept significantly less during each of the 5 nights than the flies fed drug-free food.

Evidence from both mammals and *Drosophila* suggests a role for dopamine signaling in the effect of caffeine on arousal^21^. However, these studies implicate dopamine receptors, which in mammals can interact with adenosine receptors, and thus do not unambiguously indicate a requirement of dopamine^22^. We tested the requirement of dopamine in the caffeine response using a transgenic fly line deficient for neural tyrosine hydroxylase (DTH), the rate-limiting enzyme in dopamine biosynthesis. DTH, encoded by the *pale* (*ple*) gene, is required in peripheral tissue during development, resulting in late embryonic lethality of a *ple* null mutant^23^,^24^,^25^. A nervous system-specific DTH mutant was created by expressing a DTH transgene containing a double frameshift mutation (DTH^FS±^) in a *ple* null mutant background. This DTH^FS±^ transgene produces an active form of DTH in non-nervous tissues and a truncated inactive form of the enzyme in neurons^16^. In previous studies, expression of the double frameshift transgene and the control transgene was under the control of an upstream activating sequence (UAS) driven by Gal4 lines^16^,^26^. In the current work, we utilized two fly lines expressing the control or double-frameshift mutant forms of DTH from a BAC, specifically a 20 kb segment of the DTH genomic sequence, which was cloned and inserted into attP sites in a *ple*^2^ mutant genetic background (Cichewicz *et al.* manuscript in preparation). This eliminated the need for genetic crosses to create flies containing both Gal4 and UAS transgenes to drive expression of the DTH constructs, and utilizes DTH driven by endogenous regulatory elements. Importantly, the *DTH*^*FS*±^ *BAC* transgene rescued the viability defect of the *ple* mutants, although they lacked DTH and dopamine in the adult brain, as in the case of the *DTH*^*FS*±^ transgene expressed with the UAS-Gal4 system^16^. Control flies contain a wild type copy of the DTH coding sequence (*DTH BAC*) in a *ple* mutant background, rescuing expression in both the nervous system and non-neural tissue.

We measured the effect of chronic caffeine exposure on iso31, *DTH BAC*, and *DTH*^*FS*±^ *BAC* flies by concomitantly exposing these flies to caffeine-containing food and monitoring their sleep behavior for five days. As noted above, the sleep-reducing effect of caffeine was most robust and reproducible during the dark phase, so nighttime sleep was quantified for all experiments. Female iso31 flies had a dose-dependent decrease in nighttime sleep when assayed on food containing increasing concentrations of caffeine compared to drug-free food (p < 0.0001 for the effect of drug concentration by 2-way ANOVA with Bonferroni multiple comparisons; Fig. 1A). *DTH BAC* control flies also showed a dose-dependent decrease in nighttime sleep when exposed to increasing concentrations of caffeine in the food (p = 0.0008 by 2-way ANOVA with Bonferroni multiple comparisons, when compared to control flies) (Fig. 1B). *DTH*^*FS*±^ *BAC* flies, on the other hand, were resistant to the effect of caffeine on nighttime sleep, experiencing about 650 minutes of sleep per night irrespective of drug treatment (Fig. 1C). While these figures depict data for female flies, similar results were observed for males as well (data not shown).

In addition to promoting wake, caffeine lengthens circadian period in both mammals and insects^12^,^27^. We monitored rest-activity rhythms of flies in constant conditions, and found that 0.5 mg/ml caffeine lengthened the circadian period of these rhythms from 23.8 to 25 hours (p < 0.0001; 2-way ANOVA with Bonferroni multiple comparisons; Fig. 1E). Although the effect of caffeine on *DTH BAC* control flies was more modest, it was still significant (p = 0.001). The effect of caffeine on circadian period seemed to also require dopamine, because the *DTH*^*FS*±^ *BAC* flies did not display lengthened period when monitored on caffeine-containing food (p = 0.99; Fig. 1D).

### Caffeine affects dopaminergic signaling upstream of DTH

**Figure 2:**
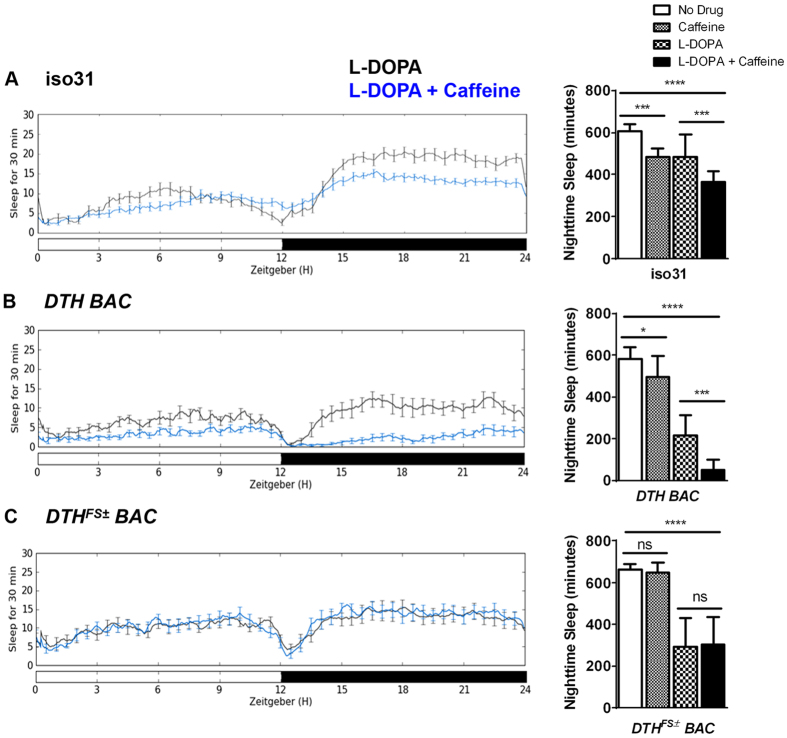
Caffeine requires dopaminergic signaling upstream of DTH. Sleep profiles for female (**A**) iso31, (**B**) *DTH BAC*, and (**C**) *DTH*^*FS*±^ *BAC* flies. Actograms depict minutes of sleep per 30 minute sliding window across a 24-hour period composed of 12 hours of light (white bar) and 12 hours of dark (black bar). Sleep is shown for flies on food containing 3 mg/ml L-DOPA (black line) or food containing 3 mg/ml L-DOPA and 0.5 mg/ml caffeine (blue line). Bar graphs quantify average number of minutes of nighttime sleep for flies of each genotype fed drug-free food (white bars), food containing 0.5 mg/ml caffeine (small check bars), food containing 3 mg/ml L-DOPA (large check bars), or food containing 3 mg/ml L-DOPA and 0.5 mg/ml caffeine (black bars). Error bars show standard deviation. See text for details of the statistical analysis.

We next sought to determine if we could rescue the caffeine response by restoring dopamine to *DTH*^*FS*±^ *BAC* mutants. DTH catalyzes the conversion of tyrosine to L-DOPA, which is then converted to dopamine by Dopa decarboxylase (Ddc)^28^,^29^. Despite lacking neural DTH, *DTH*^*FS*±^ *BAC* flies can produce dopamine if supplied with exogenous L-DOPA. Feeding L-DOPA to iso31, *DTH BAC*, and *DTH*^*FS*±^ *BAC* flies caused a sleep decrease, consistent with an augmentation of dopamine signaling in all of these genotypes (Fig. 2A–C). Both iso31 and *DTH BAC* flies experienced an even more drastic sleep loss when fed both L-DOPA and caffeine together, as compared to L-DOPA alone (Fig. 2A,B). Iso31flies slept 483 minutes per night when fed L-DOPA-containing food, and 362 minutes per night when fed food containing both L-DOPA and caffeine (p = 0.0009 by 2-way ANOVA with Bonferroni pairwise comparisons; Fig. 2A). *DTH BAC* flies slept 218 minutes per night when fed L-DOPA, and 54 minutes per night when fed L-DOPA and caffeine (p < 0.0001 by 2-way ANOVA with Bonferroni pairwise comparisons; Fig. 2B). Importantly, L-DOPA feeding did not rescue the caffeine responsiveness of *DTH*^*FS*±^ *BAC* flies (p = 0.99 by 2-way ANOVA with Bonferroni pairwise comparisons; Fig. 2C). Because rescue of dopamine synthesis downstream of DTH did not restore the caffeine response, caffeine likely modulates dopaminergic signaling upstream of DTH. However, we cannot exclude the possibility that L-DOPA-mediated rescue of dopamine synthesis in *DTH*^*FS*±^ *BAC* flies is partial and thereby insufficient for an effect of caffeine.

**Figure 3:**
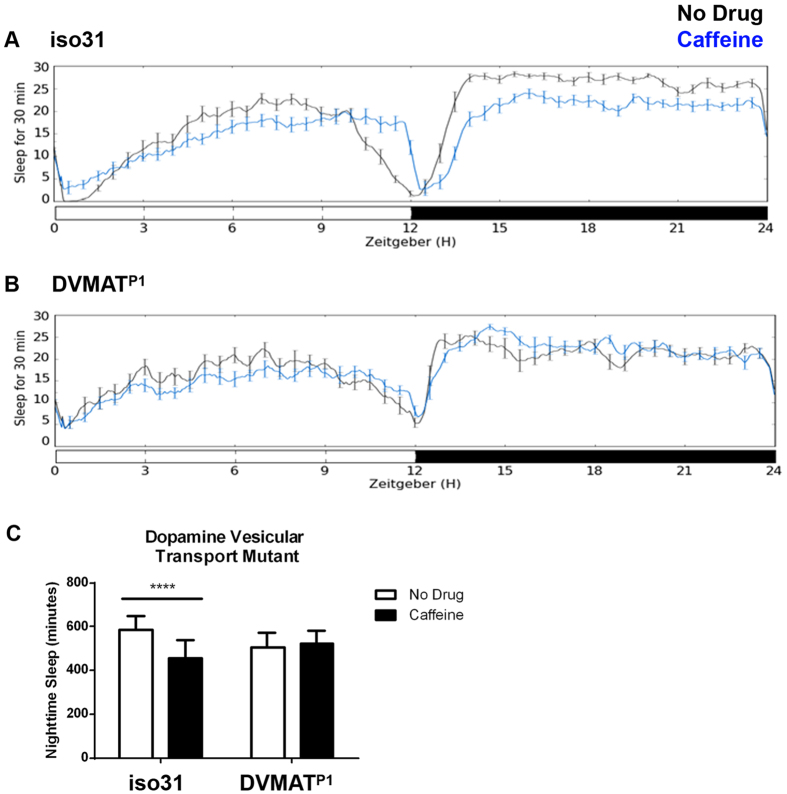
The response to caffeine requires synaptic packaging of dopamine. Sleep profiles for female (**A**) iso31 and (**B**) DVMAT^p1^ flies assayed on drug-free food (black line) or food containing 0.5 mg/ml caffeine (blue line). Graphs depict minutes of sleep per 30 minute sliding window across a 24-hour period composed of 12 hours of light (white bar) and 12 hours of dark (black bar). (**C**) Average number of minutes of nighttime sleep for flies fed drug-free food (white bars) or food containing 0.5 mg/ml caffeine (black bars). Error bars show standard deviation.

Dopaminergic signaling can be disrupted not only by blocking biosynthesis, but also by blocking synaptic release. Synaptic release of dopamine relies on the transport of dopamine into synaptic vesicles by the vesicular monoamine transporter (DVMAT). Disrupting this transport with a null mutation in the DVMAT gene blocks dopamine signaling, and we found that this mutation also blocked the wake-promoting effect of caffeine. DVMAT null mutants (*DVMAT*^*p1*^) outcrossed into an iso31 genetic background^30^ did not show any changes in night-time sleep when treated with caffeine (p = 0.86; Fig. 3).

### The PAM cluster of dopaminergic neurons is required for the response to caffeine

**Figure 4:**
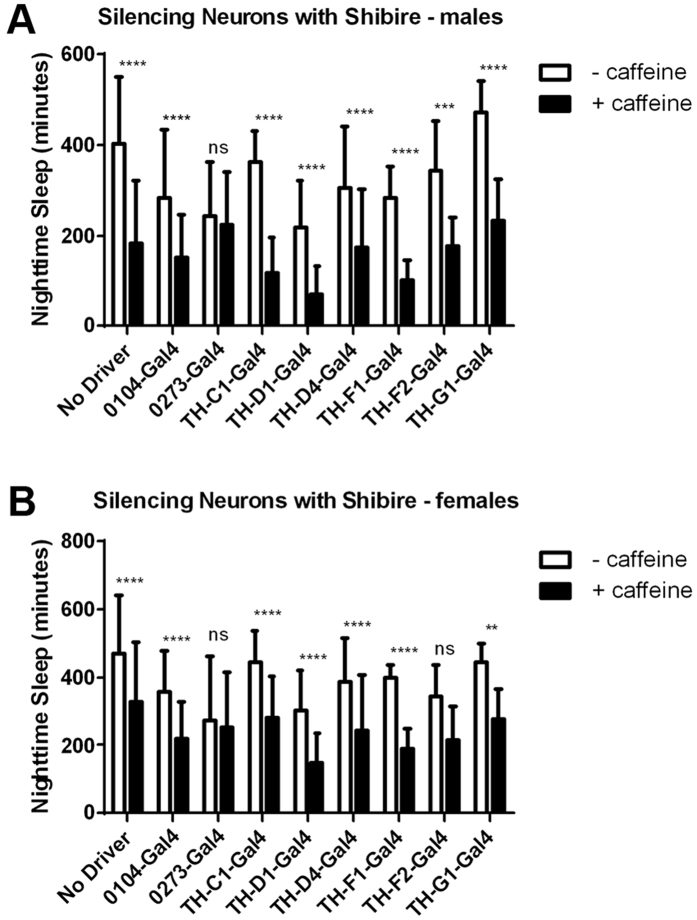
Mapping dopaminergic neurons required for the caffeine response. Average number of minutes of nighttime sleep for (**A**) male and (**B**) female flies containing various restricted dopaminergic Gal4 constructs driving expression of temperature-sensitive Shibire (UAS-Shi^ts^). Control flies contain the UAS-Shi^ts^ transgene in the absence of a Gal4 driver. Nighttime sleep is averaged across three nights at the 30 °C restrictive temperature on drug-free food (white bars) or food containing 0.5 mg/ml caffeine (black bars). Error bars show standard deviation.

There are several clusters of dopaminergic neurons in the fly brain, characterized by the location of their cell bodies and the anatomical targets of axonal projections^31^,^32^,^33^. To identify those relevant for the caffeine response, we silenced subsets of dopaminergic neurons using restricted Gal4 drivers to express the temperature-sensitive dynamin mutant Shibire^ts^ (Shi^ts^). At the restrictive temperature, 30 °C, the targeted neurons have stalled axonal transport and synaptic signaling^34^. We used six restricted dopaminergic Gal4 lines created by the Wu lab and two others from the InSite collection^35^,^36^. Six of the Gal4 lines still permitted a caffeine-induced loss of nighttime sleep at 30 °C when driving Shi^ts^. One fly line, TH-F2-Gal4 > Shi^ts^, had a significant caffeine-induced sleep reduction only in males; however, there was a strong trend towards sleep loss in females as well (p = 0.0639 by ANOVA with Bonferroni multiple comparisons). One Gal4 line, InSite0273, prevented a caffeine-induced sleep decrease when driving Shi^ts^ at 30 °C in both males and females (Fig. 4A,B). Therefore, this driver defines a group of dopaminergic neurons which, when silenced, block the wake-promoting effect of caffeine.

### Caffeine increases activity of PAM cluster neurons

**Figure 5:**
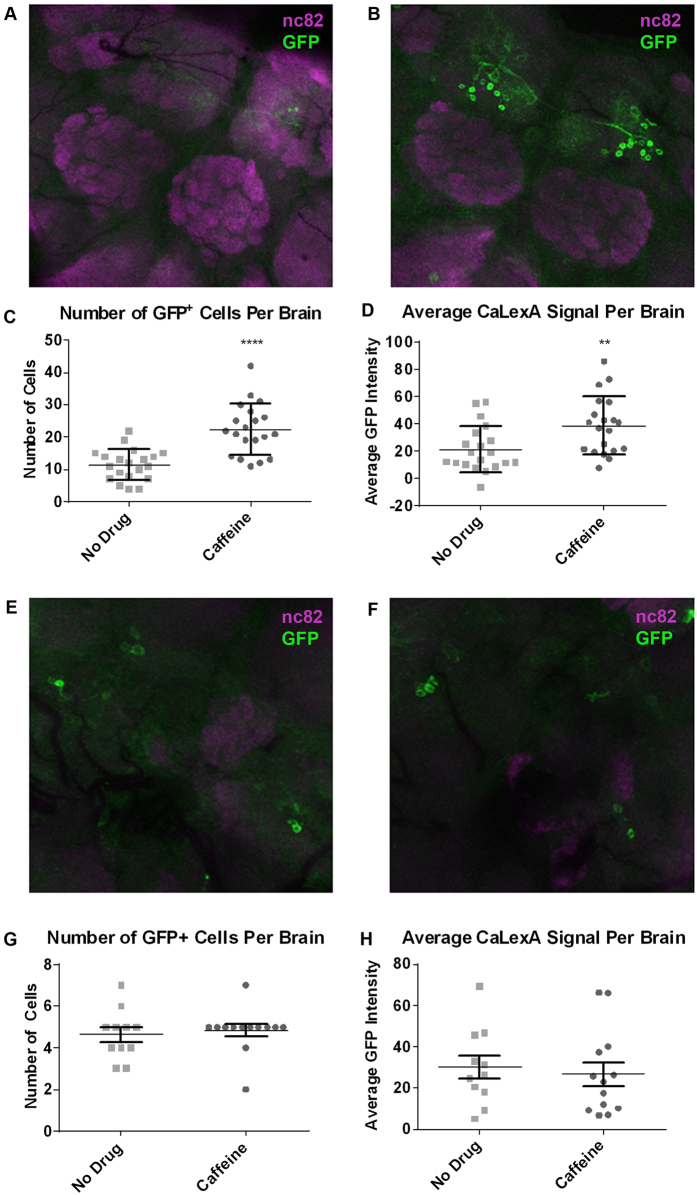
Caffeine causes increased activity of PAM cluster neurons. Immunostaining of the CaLexA signal (GFP, green) and neuropil (nc82, magenta) in brains of flies expressing the CaLexA construct under the control of the 0273-Gal4 driver to visualize PAM cluster neurons or the broader TH-Gal4 driver to visualize PPM3 cluster neurons. Flies were fed either (**A,E**) drug-free food or (**B,F**) food containing 0.5 mg/ml caffeine for 24 hours prior to dissection and staining. The GFP intensity was quantified on a cell-by-cell basis in each brain. (**C,G**) The average cell intensity for each brain is plotted for drug-free and caffeine-fed groups. (**D,H**) The number of visible GFP-positive cells in each brain is plotted for drug-free and caffeine-fed groups. Large horizontal line reflects the average, and error bars show standard deviation.

The InSite0273 driver line expresses Gal4 primarily in the Paired Anterior Medial (PAM) cluster, a group of dopaminergic neurons that projects mostly to the mushroom bodies^37^. We monitored the effect of caffeine on the PAM neurons using the CaLexA tool, in which neural activity-induced elevation of intracellular calcium results in long-term green fluorescent protein (GFP) reporter expression^19^. Representative images showed a noticeable increase in GFP fluorescence in InSite0273-Gal4 labeled cells following 24 hours of caffeine feeding (Fig. 5A,B). This increase in GFP signal was significant, both in terms of the number of cells with visible GFP expression, as well as the average GFP intensity for all of the visible cells in each brain (Fig. 5C,D). This suggests that caffeine ingestion causes increased neuronal activity in the PAM cluster neurons. The effect is not seen, however, in the PPM3 cluster, which are wake-promoting dopaminergic neurons that project to the central complex and the mushroom bodies^38^. The PPM3 neurons show no changes in CaLexA signal following caffeine feeding, showing that the effect of caffeine on activity does not extend to all dopaminergic or wake-promoting neuronal populations.

### PAM cluster neurons promote wake

**Figure 6:**
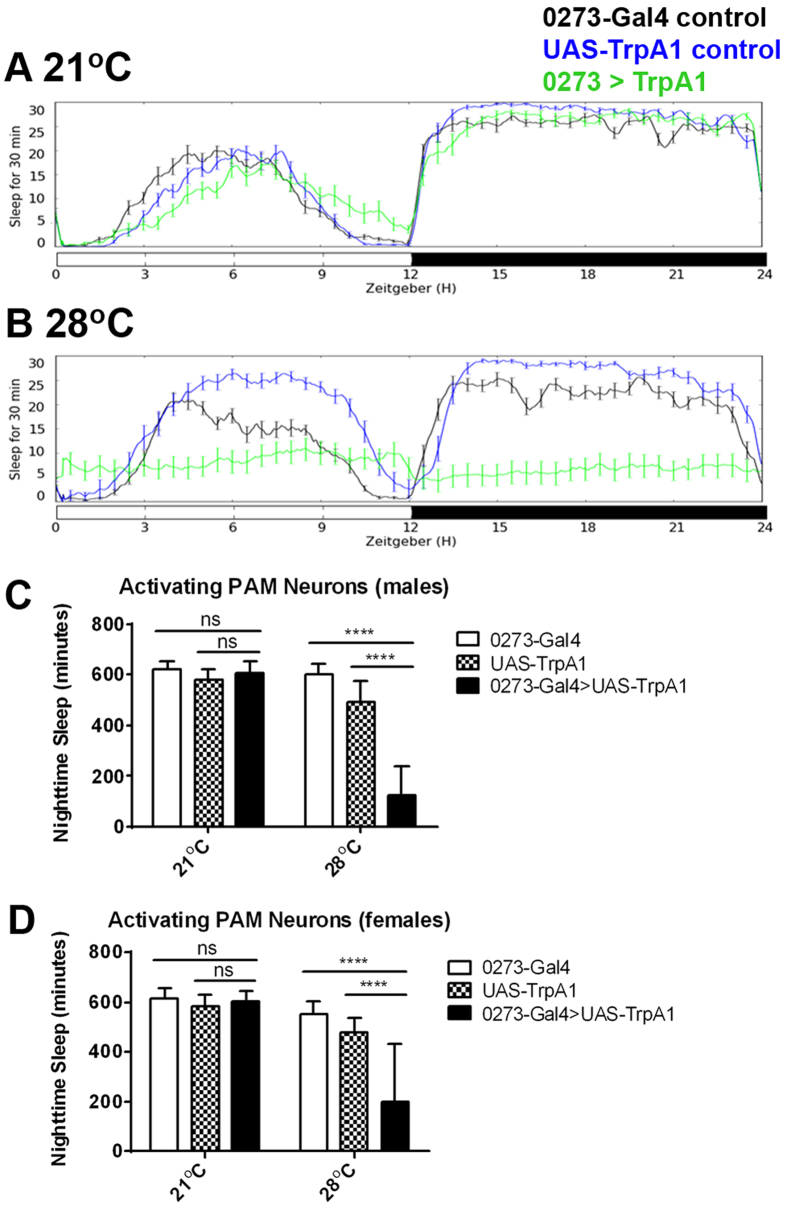
PAM cluster neurons promote wake. Sleep profiles for female 0273-Gal4 control flies (black), UAS-TrpA1 control flies (blue), or 0273-Gal4 > UAS-TrpA1 experimental flies (green) averaged across three days at (**A**) 21 °C and (**B**) 28 °C. Graphs depict minutes of sleep per 30 minute sliding window across a 24-hour period composed of 12 hours of light (white bar) and 12 hours of dark (black bar). (**C**) Average number of minutes of nighttime sleep for (**C**) male and (**D**) female flies of the three different genotypes assayed at high and low temperatures. Error bars show standard deviation.

We tested the effect on sleep of activating the PAM cluster neurons. The activity of neurons can be controlled in the laboratory setting using the heat-activated cation channel TrpA1. This channel opens in response to high temperature (28 °C), depolarizing the neurons expressing it and causing sustained activity^39^. We expressed TrpA1 under control of the 0273-Gal4 driver and assayed behavior at high and low temperatures. At 21 °C, at which temperature the TrpA1 channel is closed, experimental flies expressing TrpA1 in PAM cluster neurons had a similar sleep profile to control flies (Fig. 6A). When the behavior was assayed at 28 °C, when the TrpA1 channel is open and neurons are activated, the experimental flies showed a marked decrease in sleep which was especially evident during the nighttime (Fig. 6B). Activating PAM cluster neurons reduces sleep in both male and female flies (Fig. 6C,D), confirming that these neurons are wake-promoting.

## Discussion

Many features of human sleep are observed in Drosophila, and the fruit fly has been an invaluable tool for identifying sleep regulatory mechanisms. As in humans, caffeine treatment in *Drosophila* increases wakefulness, lowers arousal threshold, and fragments sleep^12^,^13^,^40^. While the arousal-promoting effects of caffeine are beneficial to humans during the day, they can be disruptive to sleep at night. Thus, it is important to identify all the mechanisms through which caffeine affects brain function to reduce nighttime sleep.

Most effects of caffeine, including promoting arousal, have been studied in the context of adenosine receptor antagonism. Caffeine can bind mammalian adenosine receptors, antagonizing A1 and A2a subtypes with equal affinity *in vitro* and *in vivo*^3^,^41^. Studies in mice have implicated adenosine signaling in caffeine-induced arousal, demonstrating that global or nucleus accumbens-restricted knockdown of A2A receptors blocks the response to caffeine^6^,^7^. These studies, however, only assayed the acute response, measuring wakefulness during a 3-hour window following a single injection of caffeine in a naïve mouse. This paradigm does not mimic a coffee-sipping human, nor does it account for sleep effects on a longer time scale.

The physiological and behavioral effects of caffeine seem to vary with chronic versus acute exposure and mode of administration^42^. For example, animals rapidly develop tolerance to the locomotor effects of chronic caffeine administration, but sleep reduction persists^40^,^43^. Indeed, chronic caffeine administration has very different effects and pharmacology to acute administration^44^. While adenosine receptor antagonism may be involved in acute behavioral changes following caffeine injection, other mechanisms may contribute to the prolonged effects of caffeine on sleep and arousal. Previously, the wake-promoting effect of chronic caffeine feeding was shown to be independent of the one known adenosine receptor in *Drosophila*^12^. This finding makes *Drosophila* a unique model for studying adenosine-independent mechanisms of caffeine response. In this paper, we investigate the effects of chronic caffeine exposure over the course of multiple days, focusing specifically on the role of dopaminergic signaling in the wake-promoting effect of chronic caffeine intake.

Here, we show that dopamine synthesis is required for the effect of chronic caffeine on nighttime sleep in *Drosophila*. We observed a strong and dose-dependent reduction of sleep upon caffeine treatment, and the wake-promoting effect was observed during every night of caffeine exposure. Mutants that do not produce dopamine, however, were resistant to the wake-promoting effect of caffeine. In addition to promoting wake, caffeine lengthens the period of circadian rhythms in the bread mold Neurospora crassa^45^, flies^12^, and mice^27^. We demonstrate that the dopamine-deficient *DTH*^*FS*±^ *BAC* flies are also resistant to caffeine-induced period lengthening. While the effect of caffeine on the *DTH BAC* control flies is much more modest than for iso31 flies, both genotypes show statistically significant lengthening of circadian period. The difference in magnitude of caffeine response may have to do with different expression of the endogenous DTH locus in iso31 flies versus the DTH transgene of *DTH BAC* flies. Dopaminergic signals are known to modulate some clock-controlled behaviors. Dopamine-deficient *DTH*^*FS*±^ *BAC* flies are defective in circadian entrainment and phase shifting in response to dim light cues, although the mechanisms involved have not been identified^26^.

As dopamine is a potent wake-promoting neurotransmitter, a role for it in the arousal-promoting effects of caffeine is not surprising. Previous studies in *Drosophila* showed that the dopamine receptor is required for sleep-reducing effects of caffeine^13^,^46^,^47^. As in mammals (see below), this could reflect an indirect effect, where dopamine receptors interact with other postsynaptic receptors. Our data suggest that caffeine acts presynaptically in dopamine-synthesizing neurons. Indeed, we found that L-DOPA could not restore caffeine responsiveness to *DTH*^*FS*±^ *BAC* flies. Importantly, L-DOPA was able to reduce sleep in these mutants, indicating that they contain both the neural circuitry and receptors by which dopamine promotes wakefulness. Lack of a response to caffeine is not due to a floor effect, as the *DTH BAC* control flies show a similar reduction in sleep to *DTH*^*FS*±^ *BAC* flies after L-DOPA feeding, and these controls show a further decrease with the addition of caffeine. However, we acknowledge that effects of L-DOPA could be restricted, either spatially or in terms of magnitude, thereby failing to restore the dopamine required for a caffeine response in *DTH*^*FS*±^ *BAC* flies. We also blocked effects of caffeine on sleep by perturbing release. A presynaptic effect of caffeine was supported by our finding that mutants for the vesicular monoamine transporter (DVMAT), which cannot package neurotransmitters like dopamine into synaptic vesicles, are resistant to caffeine^48^.

Caffeine has been linked to dopaminergic signaling in mammals, but always through adenosine receptors, which dimerize with and inhibit dopamine receptors^14^. Interestingly, though, dopamine levels rise in the brains of mice after acute caffeine administration, which may reflect effects of caffeine on dopamine synthesis or release^49^,^50^. In addition, increased extracellular dopamine causes caffeine hypersensitivity, as shown in both dopamine transporter (DAT) mutant mice and DAT (*fumin*) mutant flies^13^,^51^. Also, humans carrying a polymorphism associated with lower expression of the dopamine transporter are hypersensitive to caffeine, suggesting conserved mechanisms^52^.

The *Drosophila* brain contains many clusters of dopaminergic neurons, based on their locations and their anatomical targets^33^. By acutely silencing subsets of neurons, we identified the group defined by the InSite0273-Gal4 driver line, specifically the PAM cluster of neurons, as important for caffeine-induced sleep loss^37^. The PAM neurons primarily project to the medial portion and the tip of the mushroom body beta and beta prime lobes^53^,^54^,^55^. This is consistent with the finding that the DopR dopamine receptor is required in the mushroom bodies for the caffeine response^13^. Interestingly, previously-defined groups of wake-promoting neurons were dispensable in the caffeine response. Th-D4-Gal4, Th-D1-Gal4, and TH-G1-Gal4, which promote wake when transiently activated^35^, did not block the wake-promoting effect of caffeine when silenced. Therefore, the wake-promoting circuit responsible for the caffeine response seems to be distinct from previously-identified circuits.

We confirmed, using CaLexa, that the PAM cluster neurons are indeed modulated by caffeine. The InSite0273-Gal4 line expresses in all (~100–130) neurons of the PAM cluster^37^. However, only between 4 and 22 showed visible activity-dependent GFP expression at baseline, which increased to between 11 and 42 when the flies were fed caffeine. This indicates that only some PAM neurons are highly active at baseline and/or are activated by caffeine. We show that the group of PAM cluster neurons expressing the 0273-Gal4 driver are wake-promoting when activated, supporting the proposition that the activation of these cells by caffeine is the mechanism by which caffeine promotes wake. Further dissection of the PAM cluster of neurons may elucidate the identity of the caffeine-activated and wake-promoting subset(s).

Interestingly, silencing cells labeled by the InSite0104-Gal4 line, which expresses in about 40 of the PAM neurons^37^, is not sufficient to block the caffeine response. The TH-G1-Gal4 and TH-C1-Gal4 driver lines also express in some PAM cluster neurons^35^, a subset which we demonstrate are dispensable for the effect of caffeine on sleep. Studies from the Waddell lab show specialized reward functions for subsets of PAM neurons, underscoring their functional heterogeneity^37^,^56^. A subset of 15 dopaminergic neurons in the PAM cluster and the mushroom body prime lobes they innervate were recently shown to be specifically required for normal performance of the flies in a startle-induced climbing response (startle-induced negative geotaxis)^54^ as well as for the maintenance of air-puff triggered flight^57^. Additional studies will be required to characterize the neurons involved in the caffeine response within the PAM cluster.

The molecular mechanism by which caffeine increases dopaminergic signaling is still unclear, and how this alteration of dopaminergic signaling interacts with other caffeine-regulated pathways to contribute to behavioral outputs requires further study. The ability of caffeine to promote arousal requires PKA signaling in the brain; it is possible that caffeine activates PKA in dopaminergic neurons by inhibiting phosphodiesterases (PDEs)^12^,^41^,^58^. Alternatively, caffeine could stimulate dopaminergic neurons by activating ryanodine receptors, which are the major mediators of activity-induced calcium release in the cell^59^. Caffeine may also act on other cell surface receptors, or perhaps on an as-yet-unidentified adenosine receptor in the fly, as suggested by the fact that caffeine-induced sleep loss is mimicked by specific adenosine receptor antagonists in *Drosophila*^13^. We note though that an effect of caffeine on dopamine signaling could account for its positive effect on cognitive ability. An interesting question for future experiments is why the PAM cluster neurons are specifically involved in the caffeine effect. These neurons may express higher levels of sleep-relevant caffeine targets, or may simply be the only sleep-modulating neurons among caffeine-sensitive cells. While the direct molecular target is still unknown, we report the novel finding that caffeine promotes wake by activating a subset of dopaminergic neurons.

## Additional Information

**How to cite this article**: Nall, A. H. *et al.* Caffeine promotes wakefulness via dopamine signaling in *Drosophila. Sci. Rep.* **6**, 20938; doi: 10.1038/srep20938 (2016).
